# Relationship Between Brain Lesions in Patients with Post-Stroke Aphasia and Their Performance in Neuropsychological Language Assessment

**DOI:** 10.3390/neurosci6040122

**Published:** 2025-12-01

**Authors:** Jorge Romero-Castillo, Miguel Ángel Rivas-Fernández, Benxamín Varela-López, Susana Cid-Fernández, Santiago Galdo-Álvarez

**Affiliations:** 1 Department of Psychobiology and Behavioral Sciences Methodology, Universidad de Málaga, 29010 Málaga, Spain; jorgerc@uma.es; 2 Unit of Cognitive Neuroscience and Aphasia (UNCA), Centro de Investigaciones Médico-Sanitarias (CIMES), Universidad de Málaga, 29010 Málaga, Spain; 3 Area of Developmental and Educational Psychology, Department of Psychology, Sociology and Philosophy, University of León, 24071 León, Spain; mrivf@unileon.es; 4 Department of Clinical Psychology and Psychobiology, Universidade de Santiago de Compostela, 15782 Santiago de Compostela, Spain; benxamin.varela.lopez@usc.es; 5 Applied Cognitive Neuroscience and Psychogerontology Research Group, Institute of Psychology, USC (IPsiUS), 15782 Santiago de Compostela, Spain; susana.cid@usc.es; 6 Cognitive Neuroscience Research Group, Health Research Institute of Santiago de Compostela (IDIS), 15706 Santiago de Compostela, Spain; 7 Department of Developmental and Educational Psychology, Universidade de Santiago de Compostela, 15782 Santiago de Compostela, Spain

**Keywords:** post-stroke aphasia, Boston Diagnostic Aphasia Examination, Network Cortical parcellation, white matter disconnection

## Abstract

Several recent studies have utilized neuroimaging to delineate the localization and function of brain regions involved in language. However, many uncertainties persist regarding the organization of the linguistic system in the human brain. The aim of the present study was to characterize the structural changes produced in a sample of 9 patients with post-stroke aphasia (4 women; mean age = 60 years, SD = 14.86) and their relationship with performance in the entire Boston Diagnostic Aphasia Examination (BDAE). Magnetic Resonance Imaging was acquired from the brain of each patient and brain lesions were assessed. Disconnection’s severity of each white matter tract by embedding the lesion into the streamline tractography atlas of the Human Connectome Project was analyzed, and grey matter lesion load using a 7-Network Cortical parcellation template was estimated, with additional subcortical, cerebellar and brainstem parcels. Finally, all data obtained was correlated with performance in the BDAE. Somatomotor network correlated with repetition scale. The disconnection of the left acoustic radiation and inferior longitudinal fasciculus correlated with repetition sub-scale. Finally, the left U-fibers correlated with severity (a BDAE sub-scale that assesses the patient’s communicative skills), conversational speech and reading sub-scales. These findings emphasized that the disconnection of these fronto-parieto-temporal structures correlate with deficits in repetition, beyond the classical hypothesis attributing such deficits solely to the impairment of the arcuate fasciculus.

## 1. Introduction

Aphasia is a disorder resulting from a brain injury that leads to difficulty or inability to use previously acquired language skills (oral, written, and/or gestural). Among the various pathologies associated with aphasia, stroke is the most frequent in adults [1]. Stroke occurs when blood flow to the brain structures decreases or is interrupted; if it exceeds 5 min, it causes interference with normal neuronal metabolism, leading to ischemic necrosis [2] (p. 46). The risk of experiencing a stroke has increased by 50% in the last 20 years: more than 10 million people suffer from it worldwide every year, and one in four people will experience it in their lifetime [3]. Stroke is the leading cause of acquired disability in adults, the second leading cause of death, and the third cause of death and disability combined worldwide [4]. Between 21% and 38% of survivors who experience a stroke in the left hemisphere end up with aphasia [5], known also as Post-Stroke Aphasia (PSA). The lesions that most frequently cause PSA (over 80%) affect perisylvian gray matter and internal structures such as the basal nuclei (traditionally known as basal ganglia), internal capsule, or periventricular white matter, irrigated by the middle cerebral artery. However, it is less common to experience aphasic syndromes if areas between the middle cerebral artery and the anterior or posterior cerebral artery are affected, away from the Sylvian fissure [1].

Recovery of language skills after stroke can occur spontaneously and rapidly in the first months, depending on factors such as lesion size, affected brain regions, and premorbid individual variables [6,7]. Nevertheless, many individuals with PSA continue to experience persistent deficits during the chronic phase [8,9]. The traditional diagnostic framework, rooted in the 19th-century Wernicke–Lichtheim model, evaluates fluency, repetition, and comprehension to classify aphasia into eight syndromes, each linked to specific lesion sites [5,10]. However, this model has been criticized for oversimplifying the complexity of language processing, relying on imprecise constructs, and lacking consistent Lesion–Symptom correspondence [11,12,13,14]. Moreover, a significant number of patients do not fit neatly into these categories, challenging its diagnostic utility [15,16].

A stroke not only affects the function of cortical structures damaged by the lesion but also alters the functioning of distant cortical regions [17,18,19]. Therefore, it has been suggested that the disconnection of functional networks due to white matter tract damage emerges as a key mechanism leading to neurophysiological changes in regions distant from a focal lesion [20]. In other words, focal structural damage limits the strength of the connection with remote cortical regions by disrupting functional connectivity, resulting in additional behavioral deficits despite seemingly intact regions [21,22]. Since disconnection is not evident with cortical necrosis and is a complementary and independent factor explaining behavioral deficits [23], an increasing number of studies are using neuroimaging data and techniques to assess structural disconnection of language areas due to stroke-induced white matter damage [20,22,23,24,25,26,27,28,29,30,31,32,33,34] and also the estimated impact on functional connectivity [35,36,37,38,39,40]. Although individual variability and structural plasticity pose challenges for mapping the connectome (for more information about connectome mapping, see [41]), the Human Connectome Project (HCP) has conducted multiple high-resolution spatial MRI studies to more accurately delineate the cerebral connectome [42].

Further understanding of the damage caused by a stroke in the complex language processing systems is still necessary. Significant studies have suggested moving beyond classical models (based on Broca and Wernicke) as they are insufficient to fully explain aphasias [43,44]. The model proposed by Hickok and Poeppel [45,46] posits that the dorsal stream, which connects the posterior temporal lobe with frontal regions via the arcuate fasciculus, is involved in the mapping of auditory representations onto articulatory motor plans. This pathway is considered essential for repetition, particularly of pseudowords, which lack semantic content. In contrast, the ventral stream primarily supports speech recognition and language comprehension, functioning as a pathway that maps phonological (auditory) representations onto lexical-semantic representations. For this reason, the repetition of real words, which engages semantic processing, also recruits the ventral pathway, which connects the middle and anterior temporal lobe with inferior frontal regions, thereby facilitating access to meaning [47]. But the association between lesions in the ventral stream and language comprehension deficits is more complex than traditionally assumed. Evidence from Lesion–Symptom mapping and intraoperative stimulation studies indicates damage to ventral white matter tracts, such as the inferior fronto-occipital fasciculus (IFOF), inferior longitudinal fasciculus (ILF), and uncinate fasciculus, disrupts semantic processing tasks beyond simple word comprehension, affecting both verbal and non-verbal semantic associations [48]. Furthermore, lesions in anterior temporal and inferior frontal regions, particularly the pars triangularis (area 45), have been linked to impairments in semantic control—the ability to flexibly retrieve and manipulate semantic information based on context [49].

To broaden the focus on the substrates involved in aphasias and improve knowledge in the literature, the main goal of this study is to characterize the relationship between performance on the Boston Diagnostic Aphasia Examination (BDAE; [50]) tests and sub-scales and structural brain changes in nine patients with chronic post-stroke aphasia. For this purpose, we assessed the extent of stroke damage to white matter tracts using the HCP atlas as a template. This atlas contains normative maps of the human brain [51] and has been applied in previous work with post-stroke aphasia patients [26,39]. By integrating a normative reference map of brain regions that have suffered damage, it is used as a comparative method to quantify a lesion. Finally, we evaluated the impact of stroke on the integrity of regions supporting resting-state cortical networks and subcortical, cerebellar, and brainstem areas [52,53]. Based on previous classic studies on aphasia, our hypotheses are that lesions in the dorsal stream of the left perisylvian white matter will produce deficits in repetition (particularly of pseudowords) associated with a dysfunction in the mapping of acoustic signals necessary for articulation; and lesions in the ventral stream will produce deficits in comprehension associated with a dysfunction in the processing of speech signals, and could contribute to producing deficits in word repetition [5,45,46].

However, we propose that quantifying damage across distributed brain networks and all major white matter tracts, rather than focusing solely on discrete lesion locations, provides a more comprehensive and functionally informative approach to characterizing language deficits. This network-based perspective enables a finer-grained understanding of how disconnection syndromes and disruptions in structural connectivity contribute to the multifaceted impairments observed in post-stroke aphasia. Notably, this study introduces a novel methodological approach by integrating high-resolution normative data from the HCP to map and contextualize stroke-induced lesions, enhancing anatomical precision and facilitating more accurate inferences about structure-function relationships. To the best of our knowledge, this is the first MRI-based investigation to combine such a detailed connectomic framework with performance on a comprehensive neuropsychological language battery like the BDAE. Unlike conventional Lesion–Symptom mapping studies that often rely on binary classifications of cortical damage and categorical aphasia syndromes, our approach allows for continuous, quantitative comparisons of disconnection patterns with a wide array of linguistic functions assessed by the BDAE. This represents a shift toward a more nuanced, network-oriented understanding of the neural bases of language and their vulnerability in stroke.

## 2. Materials and Methods

### 2.1. Participants

**Figure 1:**
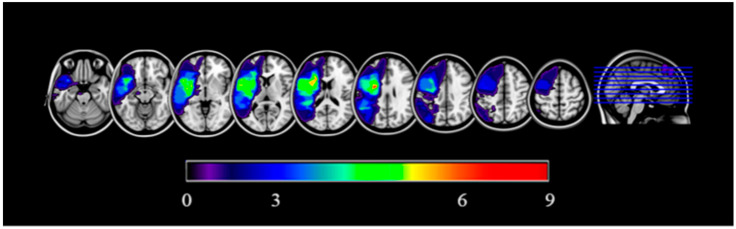
Participant’s common lesion site. Left hemisphere is presented on the left side for each axial section. Cold colors represent voxels that correspond to damaged areas in a smaller number of participants (e.g., dark purple = 1 participant with a lesion involving those particular voxels). Hot colors represent those voxels corresponding areas in a larger number of participants (e.g., red = 9 participants with a lesion involving those particular voxels). For presentation purposes, a slight smoothing was applied on the image (3 mm FWHM).

The sample consisted of 9 patients (4 females) with chronic post-stroke aphasia (whose ischemic episode, compatible with left middle cerebral artery affection, had occurred at least 6 months before participating in the study). Exclusion criteria were prior history of seizures or epilepsy, current use of anticonvulsant medications, history of cardiovascular or respiratory disease or other severe medical conditions, pre-existing dependency before stroke, contraindications for magnetic resonance imaging (e.g., metallic implants, pregnancy, claustrophobia), peripheral neuropathy or chronic pain disorders, lack of informed consent, high suicide risk, electroconvulsive therapy within the preceding six months, age over 85 years, and absence of naming errors according to the Boston Naming Test. Figure 1 illustrates the common lesion sites of the participants included in this work. The mean age was 60 years (SD = 14.86), and the years of education were 17 (SD = 4.74). Sociodemographic data, type of aphasia, and main lesion locations for each participant can be found in Table 1.

The research had the approval of the Regional Research Ethics Committee, including informed consent from patients and their families, and adhered to the principles of the Declaration of Helsinki.

### 2.2. Neuropsychological Assessment

For the neuropsychological assessment of various language components, as well as for issuing a specific diagnosis of aphasia, participants completed the Spanish version of the comprehensive Boston Diagnostic Aphasia Examination [50]. Percentiles for the following sub-scales were derived based on the participants’ percentiles achieved in the 43 subtests that make up the scale: Severity, Fluency, Conversation, Oral Comprehension, Articulation, Recitation, Repetition, Naming, Paraphasia, Reading, and Writing. In addition, 3 global indices were obtained: Language Production, Language Comprehension, and Language Competence. Individual and group scores are presented in Table 2.

### 2.3. MRI Imaging and Data Analysis

Magnetic resonance imaging was performed on a Philips 3T Achieva scanner (Philips Medical System. Best, The Netherlands). For structural MRI analysis, a sagittal T1-weighted 3D Magnetization Prepared Rapid Acquisition Gradient Echo (MPRAGE) sequence (repetition time/echo time = 7.45 ms/3.40 ms. flip angle = 8°; 180 slices, voxel size = 1 × 1 × 1 mm, field of view = 240 × 240 mm^2^, matrix size = 240 × 240 mm) was acquired.

#### 2.3.1. Lesion Data: Binary Lesion Maps Estimation

Brain lesion of each participant was automatically estimated from T1-weighted images using the Lesion Identification with Neighborhood Data Analysis (LINDA) package for R version 0.5.0. [54]. This chronic stroke segmentation method demonstrated better accuracy metrics respect to other fully automated approaches [55]. The predicted binary lesion maps obtained with LINDA segmentation were visually inspected and manually corrected (when necessary) with the Freeview tool implemented in FreeSurfer 6.0 software (http://surfer.nmr.mgh.harvard.edu/ (accessed on 1 July 2025)).

#### 2.3.2. Gray Matter Lesion Load and White Matter Disconnections

An atlas-based approach was employed to evaluate the parcel-level grey matter lesion loads and the white matter disconnections in each aphasic patient. Preprocessing involved the spatial normalization of T1-weighted images towards the Montreal Neurological Institute (MNI) using the symmetric diffeomorphic normalization method (SyN) implemented in the Advanced Normalization Tools (ANTs) version 2.3.5. [56,57]. Then, deformation fields obtained in the MNI registration were applied over native binary lesion maps using the nearest neighbor interpolation method to assure that voxels located in damaged tissue (value 1) that falls in between two new voxels (value 0 and 1) will not split its values to have some value in between (i.e., value 0.5) but keep its original value (value 1). Resulting normalized T1-weighed images and binary lesion maps have 1 mm^3^ voxel dimensions and standard MNI template image dimensions of 182 × 218 × 182.

Grey matter lesion load and white matter disconnection of each participant were estimated from the normalized binary lesion maps in MATLAB R2019a (Mathworks. Inc. Sherborn, MA, USA) using the Lesion Quantification Toolkit [58]. This software quantifies the structural impacts of focal brain lesions using an atlas-based approach to estimate the parcel-level grey matter lesion loads and several white matter disconnection severity measures. Lesion loads are estimated via a region-based damage approach according to which lesion load is estimated from the percentage of voxels in each parcel that are within the lesion [21,58].

For white matter lesions, the disconnection severity of each tract is estimated by embedding the lesion into the streamline tractography atlas of the Human Connectome Project (HCP-842 atlas) as a Region Of Interest (ROI). Then, the toolbox quantifies the percentage of streamlines of each tract whose trajectories intersect the volume occupied by the lesion [21,58].

For the grey matter, we estimated the lesion load of each participant using as reference the 400 parcel resolution template included in the toolkit that contains an augmented version of the 7-Network Cortical Parcellation template of Yeo et al., [53] together with additional subcortical and cerebellar parcels from the Automated Anatomical Labeling (AAL) atlas [52] and a brainstem parcel from the Harvard-Oxford Subcortical atlas (https://fsl.fmrib.ox.ac.uk/fsl/fslwiki/Atlases (accessed on 1 July 2025)).

### 2.4. Statistical Analysis

In order to evaluate how lesion loads in each brain network and disconnections of brain white matter tracts are associated with cognitive performance in BDAE, we conducted Pearson correlations between the white matter severity disconnection of each HCP tract and the lesion load estimated in each Yeo network with the scores obtained in the BDAE subscales and indices. This approach was used given the limited sample size and the high number of predictors. More precisely, due to the high number of brain tracts and networks evaluated, the obtained *p*-values were adjusted using the Bonferroni method and the significance level was set at *p* < 0.05.

## 3. Results

### 3.1. Grey Matter Lesion Load and White Matter Disconnection

**Figure 2:**
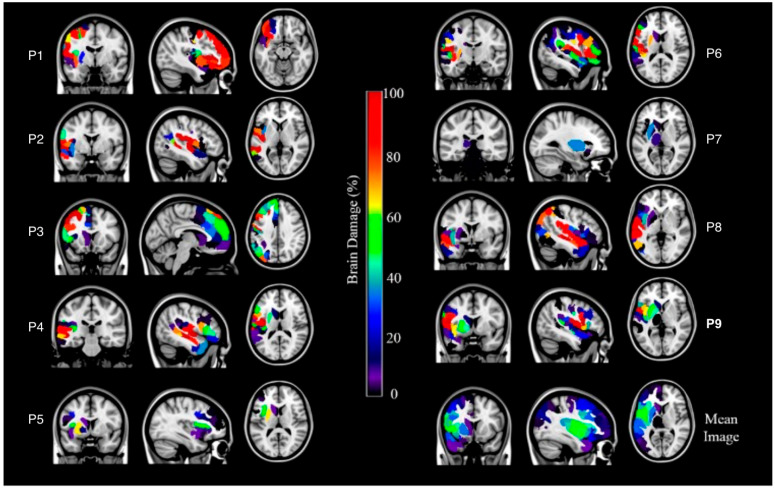
Percentage of brain damage according to the Yeo et al. [53] cortical network parcellation, the subcortical areas, the brainstem, and the cerebellum parcellations in each aphasic patient, as well as in the mean image of all patients. The left hemisphere is presented on the left side for each axial and coronal section. The sagittal view represents the left hemisphere.

Regarding the parcellated brain networks (see [53] for the coordinates of the structures participating in each of the 7 networks) (Table 3 and Figure 2), results showed that the three most affected brain networks were the Ventral Attentional Network, the Somatomotor Network, and the Default Mode Network. The Control, Dorsal Attentional, Limbic and Visual networks were also affected although to a lesser extent (less than 10%). Moreover, results revealed that the subcortical structures most affected were pallidum followed by the thalamus, the putamen, and the caudate nucleus. Residual brainstem damage was present in one aphasic patient.

Finally, WM disconnections are presented in Table 4; the disconnection of the tracts, ranked in descending order, is as follows: Arcuate Fasciculus, Inferior Fronto Occipital Fasciculus, Extreme Capsule, Frontopontine Tract, Middle Longitudinal Fasciculus, Frontal Aslant Tract, Anterior Commisure, Acoustic Radiation, Corticospinal Tract, Corticostriatal Pathway, Superior Longitudinal Fasciculus, Corticothalamic Pathway, Temporopontine Tract, Occipitopontine Tract, Parietopontine Tract, Corpus Callosum MidAnterior, Corpus Callosum Central, U-fibers, Inferior Longitudinal Fasciculus, Uncinate Fasciculus, Corpus Callosum Posterior, Corpus Callosum Anterior, Optic Radiation, Corpus Callosum MidPosterior, Cingulum, Medial Lemniscus, Spinothalamic Tract, Superior Cerebellar Peduncle, Vertical Occipital Fasciculus, and Middle Cerebellar Peduncle. No WM disconnection was found in the following brain tracts: Posterior Commisure, Fornix, Cerebellum, Inferior Cerebellar Peduncle, Vermis, Central Tegmental Tract, Dorsal Longitudinal Fasciculus, Lateral Lemniscus, Medial Longitudinal Fasciculus and the Rubrospinal Tract.

### 3.2. Correlation Analysis

Pearson correlations between the lesion load estimated in each Yeo network with the scores obtained in the BDAE subscales and indices presented only one significant correlation (once corrected by Bonferroni). Concretely, it has been found a significant negative correlation between the lesion in the Somatomotor Network and performance in the Repetition sub-scale of the BDAE (r= −0.863; *p* = 0.021).

Pearson correlations between the white matter severity disconnection of each HCP tract and the scores obtained in the BDAE subscales and indices presented 5 significant correlations. Performance in the Repetition subscale correlated negatively with the lesion in the Acoustic Radiation (r = −0.901; *p* = 0.04) and in the Inferior Longitudinal Fasciculus (r = −0.948; *p* = 0.00396). In addition, lesion in the U-fibers correlated negatively with performance in three sub-scales of BDAE: Severity (r = −0.961; *p* = 0.00148), Conversation (r = −0.909; *p* = 0.04), and Reading (r = −0.905; *p* = 0.04). Further details, including the correlation coefficients and *p*-values for all correlations, can be found in Supplementary Table S1.

## 4. Discussion

The present study aimed to characterize the relationship between performance on the Boston Diagnostic Aphasia Examination tests and sub-scales and structural brain changes in nine patients with chronic post-stroke aphasia. Most of the significant results of the present study were found in relation to the repetition tests of the BDAE. In particular, a significant negative correlation was observed between the lesion load in the somatomotor network and performance on the repetition subscale, suggesting that impairments in repetition may reflect a disruption of auditory–motor integration mechanisms. Moreover, analyses of white matter tract disconnections revealed significant associations between repetition performance and lesions in the acoustic radiation and Inferior Longitudinal Fasciculus (ILF), further supporting the role of temporo-parietal pathways in phonological processing. Additionally, disconnection of short-range U-fibers showed significant correlations with the severity, conversation, and reading subscales, indicating that local cortico-cortical disconnections may also contribute to broader language and communicative difficulties. These results will now be discussed in detail to contextualize their relevance within the existing literature and theoretical frameworks on aphasia and language networks.

Traditionally, repetition deficits have been attributed to damage to the Arcuate Fasciculus (AF), which directly and indirectly (mediating Geschwind’s territory) connects perisylvian cortical regions via the dorsal language pathway [45,46]. According to the most widely accepted classification, repetition difficulties associated with AF injury fall under the label of conduction aphasia [59,60]. However, there is increasing evidence demonstrating the inconsistency of this taxonomy due to the heterogeneity of behavioral manifestations in post-stroke aphasia patients. Specifically, the exclusive association between auditory–verbal repetition ability and AF damage is criticized [61,62].

A significant association between the BDAE repetition sub-scale and the somatomotor network (the second most affected cortical network at the group level) was found. It is essential to note that the somatomotor network includes the primary auditory cortex (Heschl’s gyrus), as well as parts of the superior temporal gyrus and somatomotor cortex, according to the functional parcellation proposed by Yeo et al. [53]. The phonological processing required for repetition involves the primary auditory cortex, located in the posteromedial transverse part of the superior temporal gyrus, including Heschl’s gyrus [63,64]. Therefore, the correlations found could be explained as a deficit in auditory processing following damage to Heschl’s gyrus and impairment of the cognitive processes necessary for auditory-motor integration, primarily supported by the left superior temporal gyrus. Additionally, it has been proposed that repetition deficits can be seen as a deterioration of sensorimotor (auditory) integration associated with damage to a specific area in the posterior part of the left superior temporal gyrus called the planum temporal [65]. The planum temporal, with its asymmetry between hemispheres, provides the neural basis for the auditory-phonological trace necessary for guiding speech production. Damage to the left hemisphere’s planum temporal affects this integration, disrupting auditory feedback and impacting repetition ability, as demonstrated in a previous magnetic resonance imaging study in post-stroke aphasia patients [66]. These findings are consistent with previous studies indicating that tasks involving repetition are anatomically supported by various perisylvian cortical regions [67]. Therefore, the impairment in the somatomotor network could be related to the deficit in auditory-motor integration necessary for verbal repetition. But this finding should be interpreted with caution. On one hand, the correlation might reflect the involvement of more general mechanisms of audio-motor integration or articulatory monitoring. On the other, the lack of correlations with regions traditionally linked to repetition, such as posterior parietal areas, raises questions about the functional specificity of this result [68]. Given that this was the only significant association observed in cortical networks, the possibility of a spurious finding cannot be ruled out.

Our results also align with recent studies applying Voxel-based Lesion–Symptom Mapping (VLSM, a technique used to analyze the relationship between brain lesioned areas and measures of language) in post-stroke aphasia patients. Baldo et al. [69] demonstrated that performance in repetition tasks and the auditory–verbal phonological storage component correlated with damage to the left superior temporal gyrus. Rogalsky et al. [70] identified the involvement of the inferior parietal lobule and superior temporal gyrus, including Heschl’s gyrus and the supramarginal gyrus, in the word repetition task of the BDAE. They concluded that damage to the underlying white matter does not fully explain repetition results. Ripamonti et al. [71] found an association between problems in repetition tasks and impairment in phonological processing with lesions in Heschl’s gyrus and the posterior perisylvian parietal region of the left hemisphere. Sul et al. [72] reported that damage to the corona radiata, superior longitudinal fasciculus, and left superior temporal gyrus correlated with poor recovery of repetition skills. In addition, Døli et al. [73] revealed that difficulties in repetition in an acute-phase patient sample were associated with lesions in the left superior temporal gyrus.

As evident from neuroimaging studies in post-stroke aphasia individuals, the classical lesion model of Wernicke–Lichtheim formulated in the 19th century, and later revised by Geschwind [60], needs updating [44]. Specifically, repetition deficits should not be exclusively explained by AF damage but rather by the injury to multiple brain structures and/or their disconnection. Focal structural damage in white matter leads to a limitation of connections between distant cortical structures, resulting in more severe behavioral deficits. In fact, our results showed that the AF is the association pathway with the highest disconnection percentage (over 90%) in the present sample. This is consistent with interpretations of previous studies supporting the disconnection hypothesis of AF as key to repetition problems. A recent study using the Connectome-based Lesion–Symptom Mapping (CLSM) approach as a complement to VLSM, to reveal anatomically crucial regions for tasks associated with white matter damage in cortical networks, has assessed the disconnection of the AF in individuals with chronic post-stroke aphasia [24]. It suggested that auditory–verbal repetition problems could be explained beyond mere AF impairment and characterized as an essential disconnection syndrome of parieto-temporal cortical regions and associated frontal circuits. They emphasized that all posterior terminations of identified white matter tracts were located in the parietal or temporal cortex, emphasizing the importance of posterior perisylvian cortical regions for sensory-motor integration necessary for verbal repetition of auditory stimuli. Associations between repetition deficits and multiple fronto-parieto-temporal disconnections, linked to AF and left superior temporal gyrus damage, are also reported in another recent study applying CLSM and VLSM with a large sample of post-stroke aphasia patients [31].

It is important to note that a review of functional neuroimaging studies in healthy individuals and patients with post-stroke aphasia has demonstrated that linguistic tasks involve cortical networks encompassing fronto-parietal regions associated with attentional and executive functions, as well as the domain-general cingulo-opercular network, specifically the dorsal ACC/SFG (anterior cingulate cortex/superior frontal gyrus), and the Default Mode Network [74]. These interpretations align with the notion of fronto-parieto-temporal damage and/or disconnection associated with repetition difficulties reported in CLSM and VLSM studies, and with the three most affected brain networks found in our study.

Additionally, we found that the second association fiber with the highest disconnection percentage (over 90%) is the Inferior Fronto-Occipital Fasciculus (IFOF), which runs along the ventral language pathway and has been proposed to participate in the lexical-semantic processing [45,46,75], but the interpretation of the IFOF’s role in language yet to be determined [76]. Given the absence of clinical semantic deficits in the present sample and the lack of significant correlations between the IFOF and performance on the BDAE repetition sub-scale, caution must be exercised in attributing a functional implication to this tract. The current data do not allow us to establish a clear relationship between the observed structural disconnection of the IFOF and any specific linguistic domain. Nevertheless, on one hand, the ventral pathway has been observed to participate in creating and identifying memory traces necessary for repetition, even without associated meaning [77,78]. On the other hand, it has been demonstrated that the redundancy of connections between cortical structures provides flexibility to the linguistic system and opens doors to compensation mechanisms between pathways after brain damage [78]. In other words, the disconnection of the dorsal pathway could lead to a redistribution of functions, and the ventral pathway could assume the repetition capacity, albeit with lower performance (or unsuccessful), even in the absence of semantic content. In this regard, given the significant disconnection observed in both dorsal and ventral tracts in this study, it is plausible that compensatory mechanisms were not sufficient to sustain adequate repetition performance.

Continuing with the contemporary dual-route model, a crucial associative tract running along the ventral pathway is the ILF [79]. The ILF comprises fibers connecting the temporal pole (anterior end of the temporal lobe) to the occipital lobe (a cortical region not directly involved in language) [80,81]. In this study, a statistically highly significant correlation was found between the disconnection of the ILF and the BDAE repetition sub-scale, contradicting the information proposed by the dual-route model regarding the linguistic functions attributed to the dorsal and ventral routes [82]. It has been suggested that the ILF’s involvement in semantic processing occurs indirectly and is not essential for language, as neither its surgical resection nor its stimulation alters semantic processing [79,83]. However, more recent findings indicate that this pathway plays a significant role in language comprehension [84]. Although knowledge about the ILF’s functional role in language processing is still limited, our results are inconsistent with the existing literature and add uncertainty regarding the ILF’s participation in language, but it is necessary to note that our interpretations could be unstable due to the low number of our sample.

Statistically significant correlations were also found between the BDAE repetition sub-scale and the disconnection of the acoustic radiation, which is a small anatomical sensory pathway transporting auditory information between the thalamus and the primary auditory cortex. While the literature does not clearly detail the role of this white matter tract in language, our results suggest that the acoustic radiation may play a role in analyzing acoustically processed signals and their integration in the thalamus to support the motor function necessary for auditory–verbal repetition [85].

Besides the effects of brain disconnection on repetition performance, statistically significant correlations were found between the disconnection of U-fibers and the severity, conversational speech, and reading sub-scales. Most U-fibers connect adjacent intrahemispheric cortical structures and run along cortical sulci [86]. Regarding language, U-fibers connecting the superior temporal gyrus with the medial temporal gyrus have been identified as an indirect pathway for auditory information, connecting the primary auditory area (Heschl’s gyrus) with the inferior frontal gyrus, mediated by the long segment of the AF [87]. However, beyond these investigations, there is not enough data in the literature to allow us to discuss these correlations, and it is expected that future studies with larger samples will allow for a more robust and well-founded discussion of the results.

Although the present analysis focused on the significant correlations identified, it is also relevant to address the lack of significant associations between other BDAE subscales and brain regions traditionally linked to their respective linguistic functions. For instance, no clear correlations were observed between comprehension-related measures and the temporal or inferior parietal regions typically associated with semantic and auditory processing in classical models of language. Likewise, naming and fluency subscales did not show consistent relationships with frontal or anterior temporal structures, despite their established involvement in lexical retrieval and speech production [5,10]. These findings may reflect the limitations of the present study.

This study has several limitations that future studies should consider. First, the use of a small sample of only nine post-stroke aphasia patients, with heterogeneous lesion volumes and locations, may limit statistical power and increase variability, potentially reducing the ability to detect subtle differences or robust correlations; therefore, interpretations should be made with caution. Second, the lack of a broader neuropsychological assessment including non-linguistic domains, such as phonological working memory because is primarily associated with repetition and the processing of auditory information [88], which would have allowed for a more comprehensive characterization of cognitive deficits beyond the language network. Thirdly, employing the LINDA package requires assuming that lesion masks may be distorted due to the displacement and reorganization of brain parenchyma after a stroke. Finally, it is crucial to acknowledge that lesion studies show which specific areas of the brain are associated with particular tasks, meaning a causal relationship between lesion and function cannot be established.

On the other hand, this study has a number of strengths. To the best of our knowledge, this is the first MRI study correlating brain damage, involvement of cortical networks according to Yeo et al.’s [53] partitioning, and the disconnection percentage of white matter tracts according to the Human Connectome Project with performance on such an extensive battery as the BDAE.

## 5. Conclusions

In conclusion, the results of this study support the hypothesis that auditory–verbal repetition deficits in individuals with post-stroke aphasia appear to be associated with lesions in (and disconnections of) various temporal and perisylvian structures in the left hemisphere, whereas the disconnection of white matter tracts as U-fibers affects a variety of language components, such as speech and reading. These findings align with previous research demonstrating that damage to the left perisylvian areas and/or the disconnection of these structures (fronto-parieto-temporal) correlates with repetition deficits, taking us beyond the classical hypothesis of AF involvement being the sole cause of such deficits.

## Figures and Tables

**Table 1:** Characterization of Patients with Chronic Post-Stroke Aphasia.

Patient	Age (in Years)	Sex	Years of Education	Aphasia Type	Lesion Site (Left Hemisphere)
1	50	Male	20	Anomic	IFG, MFG, SFG, OFC, IFO, PreCG, PostCG, insula, rolandic operculum, caudate, putamen
2	40	Female	14	Conduction	PreCG, PostCG, rolandic operculum, IFO, insula, IPL, AG, supramarginal and Heschl’s gyri, STG and MTG
3	79	Female	12	Global	FG, MFG, SFG, OFC, precuneus, IFO, rolandic operculum, PreCG, PostCG, insula, ACC, MCC, SOG, MOG, SPG, IPG, AG, MTG and caudate
4	46	Male	24	Mixed transcortical	FG, inferior OFC, IFO, PreCG, PostCG, rolandic operculum, insula, Heschl’s gyrus, STG, MTG, caudate, putamen and pallidum
5	77	Male	23	Transcortical sensory	MFG, IFG, IFO, insula, caudate, putamen, pallidum
6	71	Female	14	Global	MFG, IFG, inferior OFC, IFO, rolandic operculum, PreCG, PostCG, insula, IPL, supramarginal, angular and Heschl’s gyri, caudate, putamen and pallidum
7	53	Female	20	Anomic	OFC, olfactory, insula, putamen, pallidus and thalamus
8	51	Male	14	Mixed transcortical	Rolandic operculum, insula, MOG, PostCG, SPG, IPG, supramarginal, angular and Heschl’s giry, STG, MTG and putamen
9	73	Male	12	Mixed transcortical	MFG, IFG, IFO, PreCG, PostCG, rolandic operculum, insula, Heschl’s gyrus, STG, caudate, putamen and pallidum

**Table 2:** Scores (percentiles) in the Boston Diagnostic Aphasia Examination (BDAE) of all participants (and group mean).

Category/Subtest	Patients									Group
	1	2	3	4	5	6	7	8	9	
SEVERITY	90	80	0	40	90	0	100	40	40	53.3
FLUENCY	100	80	0	7	100	0	85	52	20	49.3
Sentence length	100	100	0	10	100	0	100	30	20	51.1
Melodic line	100	40	0	0	100	0	100	25	20	42.8
Grammatical form	100	100	0	10	100	0	70	100	20	55.6
CONVERSATION	85	85	10	50	85	0	90	50	65	57.8
Simple social responses	100	100	20	100	100	0	100	20	50	65.6
Complexity index	75	53	16	0	70	0	80	35	80	45.4
ORAL COMPREHENSION	47	60	7	38	30	16	85	5	48	37.3
Word discrimination	50	70	0	70	20	30	70	12	40	40.2
Commands	60	60	10	30	50	8	100	3	100	46.8
Complex material	30	50	10	26	20	10	85	0	5	26.2
ARTICULATION	53	33	30	39	65	5	67	39	57	43.2
Non-verbal agility	30	30	50	70	15	15	50	60	60	42.2
Verbal agility	30	40	10	18	80	0	50	18	80	36.2
Articulatory agility	100	30	30	40	100	0	100	40	30	52.2
RECITATION	93	83	25	38	83	35	100	30	60	60.5
Automated sequences	70	70	20	10	70	0	100	18	50	45.3
Recitation	100	60	60	30	100	30	100	30	100	67.8
Melody	100	100	10	10	100	100	100	10	30	62.2
Rhythm	100	100	10	100	60	10	100	60	60	66.7
REPETITION	85	30	75	27	100	11	100	13	33	52.5
Words	70	20	100	18	100	12	100	15	30	51.7
Sentences	100	40	50	35	100	10	100	10	35	53.3
NAMING	63	73	20	14	50	0	87	17	38	40.2
Naming response	80	100	30	0	70	0	80	28	28	46.2
Boston Naming Test	70	80	20	25	50	0	80	12	65	44.7
Category naming	40	40	10	15	30	0	100	10	20	29.4
PARAPHASIA	86	72	15	35	64	38	80	66	71	58.6
Speech assessment	100	50	20	25	70	0	75	70	35	49.4
Phonemic	60	30	10	20	100	20	80	40	80	48.9
Verbal	70	80	5	70	40	90	45	80	40	57.8
Neologistic	100	100	30	30	100	50	100	40	100	72.2
Multiple words	100	100	30	30	10	30	100	100	100	66.7
READING	77	83	2	59	75	12	94	26	3	47.9
Writing matching	100	100	10	100	100	40	100	40	5	66.1
Number matching	40	100	0	40	100	15	100	20	5	46.7
Picture-word matching	20	100	10	100	18	12	60	60	5	42.8
Lexical decisión	100	100	0	100	100	20	100	10	5	59.4
Word recognition	100	100	0	100	100	20	100	0	10	58.9
Morphemes	100	100	0	100	100	5	100	0	0	56.1
Word reading	100	60	0	30	100	0	100	32	0	46.9
Sentence reading	50	40	0	20	70	10	100	30	0	35.6
Sentence comprehension	100	50	0	0	20	0	100	50	0	35.6
Paragraph comprehension	60	80	0	15	40	0	80	15	0	32.2
WRITING	65	88	0	62	86	9	64	26	0	44.3
Mechanics	10	100	0	40	100	0	5	45	0	33.3
Letter selection	80	30	0	50	80	0	50	5	0	32.8
Motor skills	100	100	0	100	100	20	20	100	0	60.0
Basic vocabulary	100	100	0	100	100	0	100	5	0	56.1
Regular phonetics	40	100	0	100	100	20	100	20	0	53.3
Common irreg. words	50	100	0	60	70	20	100	20	0	46.7
Written picture naming	60	70	0	45	70	10	60	10	0	36.1
Narrative writing	80	100	0	0	70	0	75	0	0	36.1
Language Production	80	90	10	19	75	0	65	56	43	48.6
Language Comprehension	47	60	7	38	30	17	85	5	48	37.4
Language Competence	63	75	8	29	53	8	75	31	45	43.0

**Table 3:** Mean and standard deviations (in brackets) of parcel damage in left brain areas that belong to each brain network according to the 7 Cortical Network parcellation [53], including a summary of the main regions involved in each network. Absolute percent damage in left subcortical areas, cerebellum, and brainstem are also reported.

Brain Damage	Patients									Group
	1	2	3	4	5	6	7	8	9	
Brain Networks (Left Hemisphere)										
Ventral Attentional (Temporoparietal junction, ventral frontal cortex)	32.23 (44.61)	27.47 (38.70)	14.02 (25.09)	39.80 (48.49)	5.29 (14.71)	44.07 (45.62)	0.03 (0.11)	28.28 (40.02)	23.80 (37.04)	23.89 (14.89)
Somatomotor (Precentral cortex (primary motor area), postcentral cortex (primary somatosensory area), auditory-related regions in the temporal lobe)	12.09 (27.45)	30.04 (43.74)	12.38 (24.24)	16.13 (32.78)	0.00 (0.01)	26.61 (37.77)	0 (0)	19.45 (36.02)	16.50 (29.04)	14.80 (10.30)
Default Mode Network (Medial prefrontal cortex, posterior cingulate cortex, precuneus)	14.49 (32.27)	16.05 (33.36)	25.74 (35.05)	15.71 (31.51)	0 (0)	7.54 (19.95)	0.01 (0.04)	26.07 (41.22)	1.02 (5.34)	11.85 (10.32)
Control (Dorsolateral prefrontal cortex, lateral parietal regions)	35.54 (46.47)	2.07 (7.72)	14.37 (28.21)	4.48 (20.41)	1.99 (5.51)	14.95 (31.77)	0.11 (0.51)	11.37 (19.67)	2.41 (6.28)	9.70 (11.24)
Dorsal Attentional (Intraparietal sulcus, frontal eye fields)	19.16 (36.79)	0.75 (3.14)	26.17 (41.13)	0.09 (0.42)	0.14 (0.68)	9.49 (26.86)	0 (0)	7.59 (24.41)	4.03 (17.01)	7.49 (9.43)
Limbic (Orbitofrontal cortex, anterior temporal áreas)	1.81 (5.65)	0 (0)	0.40 (1.39)	6.93 (14.16)	0 (0)	1.21 (2.84)	0 (0)	2.27 (5.72)	0.42 (1.31)	1.45 (2.22)
Visual (Occipital cortex, primary and secondary visual areas, occipital lobe regions)	0 (0)	0 (0)	0.98 (4.12)	0 (0)	0 (0)	0 (0)	0 (0)	1.52 (5.80)	0 (0)	0.28 (0.57)
Left Subcortical Areas										
Pallidum (Lenticular nucleus)	24.8	0.39	0.1	46.35	64.1	68.53	33.9	5.05	53.2	32.94 (26.95)
Thalamus	0	0	0	1.42	15.97	21.76	2.26	0.04	43.39	9.43 (15.08)
Putamen (Lenticular nucleus)	1.26	0	4.58	13.55	6.07	4.59	2.25	0	13.94	5.14 (5.31)
Caudate nucleus	0	0	0	0	0.09	0	9.01	0	0.07	1.02 (3.00)
Cerebellum	0	0	0	0	0	0	0	0	0	0.00 (0.00)
Brainstem	0	0	0	0	0	0	0.48	0	0	0.05 (0.16)

**Table 4:** Percentage of streamlines affected (ranked from most to least disconnected) in each white matter tract included in the Human Connectome Project atlas (HCP-842). Mean and standard deviations (in brackets) of each white matter tract in the whole sample are also reported.

White Matter Tracts (Left Hemisphere)	Patients									Group
	1	2	3	4	5	6	7	8	9	
Association Pathways										
Arcuate Fasciculus (AF)	100	70.36	100	100	83.42	100	95.04	100	100	94.31 (10.53)
Inferior Fronto Occipital Fasciculus (IFOF)	98.62	92.69	51.76	100	99.34	100	98.24	97.82	79.72	90.91 (16.04)
Extreme Capsule (EMC)	92.63	92.63	60.66	100	45.49	100	68.57	100	100	84.44 (20.73)
Middle Longitudinal Fasciculus (MdLF)	44.90	100	100	100	0	100	0	100	100	71.66 (44.45)
Frontal Aslant Tract (AST)	100	0	100	99.71	95.90	100	24.20	11.96	98.31	70.01 (43.91)
Superior Longitudinal Fasciculus (SLF)	89.39	14.99	94.38	54.26	28.03	95.81	47.22	33.03	77.92	59.45 (30.86)
U-fibers (U)	29.92	31.27	70.99	41.15	17.89	69.14	3.72	48.34	50.20	40.29 (22.27)
Inferior Longitudinal Fasciculus (ILF)	3.00	68.34	26.27	75.39	0	79.40	0	69.12	34.19	39.52 (34.00)
Uncinate Fasciculus (UF)	62.50	1.44	0	94.15	19.06	92.54	22.75	36.60	23.56	39.18 (35.88)
Cingulum (C)	4.87	0	80.90	0	0.95	0	0	0	0	9.64 (26.77)
Vertical Occipital Fasciculus (VOF)	0	0	19.74	0	0	0	0	0.12	0	2.21 (6.58)
Commisural Pathways										
Anterior Commisure (AC)	58.90	30.37	0.61	98.96	92.00	99.06	93.79	33.47	98.96	67.35 (37.67)
Corpus Callosum MidAnterior (CCMidAnterior)	83.62	0.98	99.89	38.74	72.00	44.31	6.01	4.96	42.39	43.66 (35.92)
Corpus Callosum Central (CCCentral)	65.69	0.41	88.69	53.01	7.39	51.79	75.97	0	47.91	43.43 (33.20)
Corpus Callosum Posterior (CCPosterior)	0.74	45.87	61.82	42.15	0.42	61.89	0.49	64.75	46.98	36.12 (27.82)
Corpus Callosum Anterior (CCAnterior)	60.01	0	85.32	18.31	59.27	23.32	0.19	0.13	2.88	27.71 (32.34)
Corpus Callosum MidPosterior (CCMidPost)	3.03	0.22	16.82	21.76	0	19.85	63.52	1.12	23.56	16.65 (20.17)
Posterior Commisure (PC)	0	0	0	0	0	0	0	0	0	0 (0)
Projection Pathways										
Frontopontine Tract (FPT)	91.77	0	99.92	99.92	91.19	100	94.81	0	99.84	75.27 (42.82)
Acoustic Radiation (AR)	0	97.78	81.31	91.12	0	100	16.07	100	96.31	64.73 (45.13)
Corticospinal Tract (CST)	52.22	2.02	76.82	95.33	32.63	100	100	5.50	95.61	62.24 (40.46)
Corticostriatal Pathway (CS)	79.36	13.83	79.87	74.84	81.82	80.38	61.79	15.27	63.64	61.20 (27.43)
Corticothalamic Pathway (CT)	46.32	11.80	74.75	54.59	41.65	88.51	30.86	33.66	61.41	49.28 (23.50)
Temporopontine Tract (TPT)	0	28.45	99.14	0	0	100	99.14	100	15.52	49.14 (48.70)
Occipitopontine Tract (OPT)	0	15.59	91.31	14.48	0	100	82.85	96.44	40.31	49.00 (43.26)
Parietopontine Tract (PPT)	0.35	5.85	30.63	74.17	0	98.25	97.82	23.82	75.65	45.17 (41.27)
Optic Radiation (OR)	0	8.57	37.55	6.12	0	94.29	1.22	88.57	0	26.26 (38.21)
Fornix (F)	0	0	0	0	0	0	0	0	0	0 (0)
Cerebellum										
Superior Cerebellar Peduncle (SCP)	0	0	0	0	18.52	0	19.66	0	0	4.24 (8.42)
Middle Cerebellar Peduncle (MCP)	0	0	0	0	0	0	0.06	0	0	0.01 (0.02)
Cerebellum (CB)	0	0	0	0	0	0	0	0	0	0 (0)
Inferior Cerebellar Peduncle (ICP)	0	0	0	0	0	0	0	0	0	0 (0)
Vermis (V)	0	0	0	0	0	0	0	0	0	0 (0)
Brainstem										
Medial Lemniscus (ML)	0	0	0	0	0	0	79.61	0	0	8.85 (26.54)
Spinothalamic Tract (STT)	0	0	0	0	0	0	47.69	0	0	5.30 (15.90)
Central Tegmental Tract (CTT)	0	0	0	0	0	0	0	0	0	0 (0)
Dorsal Longitudinal Fasciculus (DLF)	0	0	0	0	0	0	0	0	0	0 (0)
Lateral Lemniscus (LL)	0	0	0	0	0	0	0	0	0	0 (0)
Medial Longitudinal Fasciculus (MLF)	0	0	0	0	0	0	0	0	0	0 (0)
Rubrospinal Tract (RST)	0	0	0	0	0	0	0	0	0	0 (0)

## Data Availability

Dataset with T1 of all participants of the study are available anonymized (under Creative Commons 4.0 license) in Galdo-Alvarez (2024; https://doi.org/10.12751/g-node.lmbq21).

## Supplementary Materials

The following supporting information can be downloaded at https://www.mdpi.com/article/10.3390/neurosci6040122/s1: Table S1: Pearson correlation coefficients and corresponding *p*-values (in parentheses) between lesion load in each Yeo brain network and Human Connectome Project tract, and scores on the BDAE subscales.

## Abbreviations

The following abbreviations are used in this manuscript:

AAL	Automated Anatomical Labeling
ACC	Anterior Cingulate Cortex
AF	Arcuate Fasciculus
AG	Angular Gyrus
ANTs	Advanced Normalization Tools
BDAE	Boston Diagnostic Aphasia Examination
CLSM	Connectome-based Lesion–Symptom Mapping
FG	Fusiform Gyrus
FWHM	Full Width at Half Maximum
HCP	Human Connectome Project
IFG	Inferior Frontal Gyrus
IFO	Inferior Frontal Operculum
IFOF	Inferior Fronto-Occipital Fasciculus
ILF	Inferior Longitudinal Fasciculus
IPG	Inferior Parietal Gyrus
IPL	Inferior Parietal Lobule
LINDA	Lesion Identification with Neighborhood Data Analysis
MCC	Middle Cingulate Cortex
MFG	Middle Frontal Gyrus
MOG	Middle Occipital Gyrus
MPRAGE	Magnetization Prepared Rapid Acquisition Gradient Echo
MRI	Magnetic Resonance Imaging
MTG	Middle Temporal Gyrus
OFC	Orbitofrontal Cortex
PostCG	Postcentral Gyrus
PreCG	Precentral Gyrus
PSA	Post-Stroke Aphasia
ROI	Region Of Interest
SFG	Superior Frontal Gyrus
SOG	Superior Occipital Gyrus
SPG	Superior Parietal Gyrus
STG	Superior Temporal Gyrus
VLSM	Voxel-based Lesion–Symptom Mapping
