# Supplementary material for: Relationship Between Brain Lesions in Patients with Post-Stroke Aphasia and Their Performance in Neuropsychological Language Assessment
Source: NeuroSci. 2025 Dec 1;6(4):122. doi: 10.3390/neurosci6040122 (PMC12735612; doi:10.3390/neurosci6040122)
Supplement: Supplementary file 1 [file neurosci-06-00122-s001.zip › neurosci-3817971-supplementary.pdf]

Supplementary Table 1. Pearson correlation coefficients and corresponding p-values (in parentheses) between lesion load in each Yeo brain network and Human Connectome Project tract, and scores on the BDAE subscales.

	**Severity**	**Fluency**	**Conversation**	**Oral Comprehension**	**Articulation**	**Recitation**	**Repetition**	**Naming**	**Paraphasia**	**Reading**	**Writing**	**Language Production**	**Language Comprehension**	**Language Competence**
Brain Networks														
Visual	-0.42 (0.27)	-0.23 (0.56)	-0.38 (0.31)	-0.66 (0.05)	-0.21 (0.58)	-0.61 (0.08)	-0.23 (0.55)	-0.41 (0.27)	-0.29 (0.44)	-0.47 (0.20)	-0.44 (0.23)	-0.18 (0.64)	-0.67 (0.05)	-0.45 (0.22)
Ventral Attentional	-0.48 (0.19)	-0.47 (0.20)	-0.45 (0.22)	-0.35 (0.35)	-0.70 (0.04)	-0.48 (0.20)	-0.79 (0.01)	-0.59 (0.09)	-0.23 (0.55)	-0.32 (0.40)	-0.23 (0.55)	-0.39 (0.30)	-0.35 (0.36)	-0.42 (0.26)
Somatomotor	-0.48 (0.19)	-0.42 (0.26)	-0.41 (0.27)	-0.27 (0.48)	-0.79 (0.01)	-0.43 (0.24)	**-0.86 (0.02)***	-0.39 (0.30)	-0.20 (0.62)	-0.36 (0.34)	-0.26 (0.51)	-0.22 (0.57)	-0.27 (0.49)	-0.27 (0.48)
DMN	-0.44 (0.24)	-0.29 (0.44)	-0.41 (0.27)	-0.58 (0.10)	-0.49 (0.18)	-0.60 (0.09)	-0.36 (0.34)	-0.40 (0.29)	-0.42 (0.26)	-0.30 (0.43)	-0.23 (0.56)	-0.19 (0.62)	-0.59 (0.10)	-0.42 (0.26)
Control	-0.11 (0.78)	0.08 (0.85)	-0.19 (0.62)	-0.31 (0.42)	-0.23 (0.55)	-0.04 (0.91)	0.09 (0.82)	-0.14 (0.72)	0.04 (0.91)	-0.10 (0.80)	-0.15 (0.69)	-0.03 (0.94)	-0.31 (0.42)	-0.18 (0.65)
Dorsal Attentional	-0.46 (0.21)	-0.26 (0.49)	-0.51 (0.17)	-0.51 (0.16)	-0.33 (0.38)	-0.35 (0.36)	0.15 (0.70)	-0.28 (0.46)	-0.39 (0.30)	-0.47 (0.21)	-0.51 (0.16)	-0.33 (0.39)	-0.51 (0.16)	-0.47 (0.20)
Limbic	-0.22 (0.58)	-0.38 (0.32)	-0.18 (0.64)	-0.18 (0.65)	-0.18 (0.64)	-0.41 (0.27)	-0.40 (0.29)	-0.47 (0.20)	-0.31 (0.41)	-0.00 (1.00)	0.06 (0.88)	-0.36 (0.34)	-0.18 (0.64)	-0.32 (0.40)
White Matter Tracts														
Anterior Commisure	0.20 (0.60)	0.00 (1.00)	0.19 (0.63)	0.38 (0.32)	0.26 (0.50)	0.27 (0.49)	0.01 (0.99)	0.00 (0.99)	0.23 (0.55)	0.17 (0.65)	0.13 (0.74)	-0.11 (0.78)	0.39 (0.31)	0.13 (0.74)
Arcuate Fasciculus	-0.50 (0.17)	-0.53 (0.14)	-0.50 (0.17)	-0.36 (0.35)	-0.11 (0.77)	-0.49 (0.18)	-0.11 (0.77)	-0.55 (0.13)	-0.30 (0.44)	-0.55 (0.12)	-0.69 (0.04)	-0.64 (0.07)	-0.36 (0.35)	-0.58 (0.10)
Acoustic Radiation	-0.73 (0.03)	-0.76 (0.02)	-0.60 (0.09)	-0.39 (0.29)	-0.69 (0.04)	-0.74 (0.02)	**-0.90 (0.04)***	-0.62 (0.08)	-0.48 (0.19)	-0.65 (0.06)	-0.55 (0.12)	-0.53 (0.14)	-0.39 (0.30)	-0.53 (0.14)
C.C. Anterior	-0.17 (0.66)	-0.01 (0.97)	-0.26 (0.49)	-0.43 (0.24)	-0.03 (0.94)	-0.11 (0.78)	0.54 (0.13)	-0.15 (0.70)	-0.43 (0.25)	-0.14 (0.72)	-0.07 (0.85)	-0.18 (0.64)	-0.43 (0.24)	-0.34 (0.38)
C.C. Central	-0.26 (0.51)	-0.36 (0.34)	-0.32 (0.40)	0.13 (0.73)	-0.02 (0.96)	-0.06 (0.89)	0.36 (0.33)	-0.03 (0.94)	-0.33 (0.38)	-0.19 (0.63)	-0.40 (0.29)	-0.49 (0.17)	0.14 (0.72)	-0.24 (0.53)
C.C. MidAnterior	-0.26 (0.51)	-0.15 (0.70)	-0.30 (0.43)	-0.41 (0.27)	-0.01 (0.98)	-0.14 (0.71)	0.45 (0.23)	-0.24 (0.53)	-0.41 (0.28)	-0.29 (0.45)	-0.23 (0.55)	-0.29 (0.45)	-0.41 (0.27)	-0.39 (0.30)
C.C. MidPosterior	0.08 (0.83)	-0.14 (0.71)	0.03 (0.93)	0.56 (0.12)	0.24 (0.53)	0.20 (0.60)	0.27 (0.49)	0.26 (0.50)	0.03 (0.95)	0.12 (0.76)	-0.14 (0.71)	-0.24 (0.54)	0.56 (0.12)	0.13 (0.73)
C.C. Posterior	-0.86 (0.00)	-0.79 (0.01)	-0.77 (0.02)	-0.63 (0.07)	-0.78 (0.01)	-0.87 (0.00)	-0.82 (0.01)	-0.74 (0.02)	-0.61 (0.08)	-0.78 (0.01)	-0.69 (0.04)	-0.62 (0.07)	-0.63 (0.07)	-0.71 (0.03)
Corticospinal Tract	-0.34 (0.37)	-0.55 (0.13)	-0.36 (0.34)	0.18 (0.65)	-0.06 (0.89)	-0.14 (0.73)	0.08 (0.84)	-0.23 (0.56)	-0.34 (0.37)	-0.28 (0.47)	-0.44 (0.23)	-0.66 (0.05)	0.18 (0.63)	-0.32 (0.40)
Corticostriatal Pathway	-0.17 (0.66)	-0.22 (0.57)	-0.26 (0.49)	-0.09 (0.82)	0.07 (0.86)	-0.00 (0.99)	0.46 (0.21)	-0.20 (0.60)	-0.36 (0.35)	-0.13 (0.74)	-0.17 (0.67)	-0.46 (0.22)	-0.08 (0.83)	-0.33 (0.39)
Corticothalamic Pathway	-0.79 (0.01)	-0.76 (0.02)	-0.82 (0.01)	-0.55 (0.13)	-0.51 (0.16)	-0.61 (0.08)	-0.19 (0.63)	-0.74 (0.02)	-0.68 (0.04)	-0.74 (0.02)	-0.75 (0.02)	-0.88 (0.00)	-0.54 (0.13)	-0.83 (0.01)
Cingulum	-0.50 (0.17)	-0.40 (0.29)	-0.51 (0.16)	-0.44 (0.24)	-0.24 (0.54)	-0.43 (0.25)	0.26 (0.51)	-0.24 (0.54)	-0.67 (0.05)	-0.45 (0.23)	-0.45 (0.23)	-0.42 (0.26)	-0.44 (0.24)	-0.49 (0.18)
Extreme Capsule	-0.29 (0.45)	-0.36 (0.35)	-0.18 (0.64)	-0.03 (0.94)	-0.43 (0.24)	-0.29 (0.44)	-0.82 (0.01)	-0.33 (0.39)	0.11 (0.77)	-0.26 (0.50)	-0.27 (0.48)	-0.18 (0.64)	-0.03 (0.94)	-0.13 (0.74)
Frontal Aslant Tract	-0.38 (0.31)	-0.41 (0.28)	-0.39 (0.30)	-0.33 (0.38)	-0.08 (0.83)	-0.25 (0.52)	0.15 (0.69)	-0.46 (0.22)	-0.44 (0.24)	-0.39 (0.30)	-0.34 (0.37)	-0.54 (0.14)	-0.33 (0.39)	-0.50 (0.17)
Frontopontine Tract	-0.17 (0.67)	-0.29 (0.44)	-0.22 (0.56)	0.07 (0.85)	0.14 (0.71)	0.02 (0.95)	0.42 (0.26)	-0.14 (0.71)	-0.30 (0.43)	-0.16 (0.68)	-0.25 (0.52)	-0.49 (0.18)	0.08 (0.84)	-0.27 (0.49)
Inferior Fronto Occipital Fasciculus	0.51 (0.16)	0.47 (0.21)	0.43 (0.25)	0.32 (0.40)	0.15 (0.70)	0.40 (0.29)	-0.12 (0.75)	0.19 (0.63)	0.55 (0.13)	0.58 (0.10)	0.58 (0.10)	0.38 (0.32)	0.32 (0.40)	0.40 (0.28)
Inferior Longitudinal Fasciculus	-0.58 (0.10)	-0.60 (0.09)	-0.52 (0.15)	-0.38 (0.31)	-0.77 (0.01)	-0.66 (0.06)	**-0.95 (0.004)***	-0.62 (0.07)	-0.40 (0.28)	-0.38 (0.32)	-0.25 (0.51)	-0.44 (0.24)	-0.38 (0.32)	-0.47 (0.20)
Middle Cerebellar Peduncle	0.46 (0.22)	0.31 (0.42)	0.36 (0.34)	0.68 (0.04)	0.45 (0.23)	0.50 (0.17)	0.48 (0.19)	0.58 (0.10)	0.34 (0.37)	0.47 (0.20)	0.20 (0.60)	0.19 (0.63)	0.68 (0.04)	0.47 (0.21)
Medial Lemniscus	0.46 (0.22)	0.31 (0.42)	0.36 (0.34)	0.68 (0.04)	0.45 (0.23)	0.50 (0.17)	0.48 (0.19)	0.58 (0.10)	0.34 (0.37)	0.47 (0.20)	0.20 (0.60)	0.19 (0.63)	0.68 (0.04)	0.47 (0.21)
Middle Longitudinal Fasciculus	-0.76 (0.02)	-0.75 (0.02)	-0.62 (0.07)	-0.49 (0.18)	-0.73 (0.03)	-0.76 (0.02)	-0.86 (0.00)	-0.65 (0.06)	-0.50 (0.17)	-0.68 (0.04)	-0.57 (0.11)	-0.52 (0.15)	-0.49 (0.18)	-0.57 (0.11)
Occipitopontine Tract	-0.60 (0.09)	-0.51 (0.17)	-0.65 (0.06)	-0.36 (0.34)	-0.46 (0.21)	-0.54 (0.13)	-0.28 (0.47)	-0.41 (0.27)	-0.39 (0.30)	-0.56 (0.12)	-0.70 (0.04)	-0.56 (0.11)	-0.36 (0.34)	-0.54 (0.14)
Optic Radiation	-0.67 (0.05)	-0.47 (0.21)	-0.74 (0.02)	-0.71 (0.03)	-0.74 (0.02)	-0.70 (0.04)	-0.60 (0.09)	-0.70 (0.04)	-0.40 (0.29)	-0.58 (0.10)	-0.55 (0.12)	-0.51 (0.16)	-0.70 (0.03)	-0.68 (0.04)
Parietopontine Tract	-0.35 (0.35)	-0.56 (0.12)	-0.36 (0.34)	0.22 (0.57)	-0.20 (0.61)	-0.21 (0.58)	-0.27 (0.49)	-0.25 (0.51)	-0.23 (0.56)	-0.27 (0.49)	-0.44 (0.24)	-0.61 (0.08)	0.22 (0.56)	-0.27 (0.48)
Superior Cerebellar Peduncle	0.62 (0.08)	0.56 (0.11)	0.50 (0.17)	0.45 (0.22)	0.66 (0.06)	0.60 (0.09)	0.73 (0.03)	0.54 (0.13)	0.33 (0.39)	0.56 (0.11)	0.48 (0.19)	0.37 (0.33)	0.45 (0.22)	0.46 (0.21)
Superior Longitudinal Fasciculus	-0.57 (0.11)	-0.54 (0.13)	-0.61 (0.08)	-0.29 (0.44)	-0.35 (0.35)	-0.33 (0.38)	-0.00 (1.00)	-0.42 (0.26)	-0.40 (0.29)	-0.58 (0.10)	-0.69 (0.04)	-0.65 (0.06)	-0.29 (0.45)	-0.55 (0.12)
Spinothalamic Tract	0.46 (0.22)	0.31 (0.42)	0.36 (0.34)	0.68 (0.04)	0.45 (0.23)	0.50 (0.17)	0.48 (0.19)	0.58 (0.10)	0.34 (0.37)	0.47 (0.20)	0.20 (0.60)	0.19 (0.63)	0.68 (0.04)	0.47 (0.21)
Temporopontine Tract	-0.45 (0.22)	-0.33 (0.38)	-0.55 (0.12)	-0.28 (0.46)	-0.43 (0.25)	-0.40 (0.28)	-0.15 (0.70)	-0.25 (0.52)	-0.32 (0.40)	-0.38 (0.32)	-0.53 (0.15)	-0.41 (0.27)	-0.28 (0.47)	-0.40 (0.28)
Uncinate Fasciculus	-0.26 (0.49)	-0.34 (0.38)	-0.33 (0.38)	-0.17 (0.67)	-0.38 (0.31)	-0.27 (0.47)	-0.40 (0.29)	-0.51 (0.16)	-0.18 (0.65)	-0.06 (0.87)	-0.07 (0.85)	-0.46 (0.22)	-0.16 (0.68)	-0.37 (0.33)
U-fibers	**-0.96 (0.002)***	-0.84 (0.00)	**-0.91 (0.04)***	-0.77 (0.01)	-0.79 (0.01)	-0.87 (0.00)	-0.60 (0.09)	-0.84 (0.01)	-0.73 (0.03)	**-0.91 (0.04)***	-0.82 (0.01)	-0.78 (0.01)	-0.77 (0.02)	-0.88 (0.00)
Vertical Occipital Fasciculus	-0.52 (0.15)	-0.43 (0.25)	-0.53 (0.14)	-0.44 (0.23)	-0.25 (0.51)	-0.45 (0.22)	0.23 (0.56)	-0.26 (0.51)	-0.69 (0.04)	-0.47 (0.20)	-0.47 (0.21)	-0.44 (0.23)	-0.44 (0.23)	-0.51 (0.17)

Abbreviations: C.C. Anterior = Corpus Callosum. The following tracts were excluded from the correlation analyses because no brain lesions were detected in them: Central Tegmental Tract, Cerebellum, Dorsal Longitudinal Fasciculus, Fornix, Inferior Cerebellar Peduncle, Lateral Lemniscus, Medial Longitudinal Fasciculus, Posterior Commissure, Rubrospinal Tract, and Vermis. * Brain networks and white matter tracts showing a significant effect ($p < 0.05$) after Bonferroni correction for multiple comparisons are shown in bold.
